# Mild Joint Symptoms Are Associated with Lower Risk of Falls than Asymptomatic Individuals with Radiological Evidence of Osteoarthritis

**DOI:** 10.1371/journal.pone.0141368

**Published:** 2015-10-22

**Authors:** Sumaiyah Mat, Pey June Tan, Chin Teck Ng, Farhana Fadzli, Faizatul I. Rozalli, Ee Ming Khoo, Keith D. Hill, Maw Pin Tan

**Affiliations:** 1 Ageing and Age-Associated Disorders Research Group, Faculty of Medicine, University of Malaya, Kuala Lumpur, Malaysia; 2 Department of Rheumatology & Immunology, Singapore General Hospital, Singapore, Singapare; 3 Department of Biomedical Imaging, Faculty of Medicine, University of Malaya, Kuala Lumpur, Malaysia; 4 Department of Primary Care Medicine, Faculty of Medicine, University of Malaya, Kuala Lumpur, Malaysia; 5 School of Physiotherapy and Exercise Science, Faculty of Health Sciences, Curtin University, Perth, Western Australia, Australia; 6 Division of Geriatric Medicine, Department of Medicine, Faculty of Medicine, University of Malaya, Kuala Lumpur, Malaysia; University of Toronto, CANADA

## Abstract

Osteoarthritis (OA) exacerbates skeletal muscle functioning, leading to postural instability and increased falls risk. However, the link between impaired physical function, OA and falls have not been elucidated. We investigated the role of impaired physical function as a potential mediator in the association between OA and falls. This study included 389 participants [229 fallers (≥2 falls or one injurious fall in the past 12 months), 160 non-fallers (no history of falls)], age (≥65 years) from a randomized controlled trial, the Malaysian Falls Assessment and Intervention Trial (MyFAIT). Physical function was assessed using Timed Up and Go (TUG) and Functional Reach (FR) tests. Knee and hip OA were diagnosed using three methods: Clinical, Radiological and Self-report. OA symptom severity was assessed using the Western Ontario and McMaster Universities Arthritis Index (WOMAC). The total WOMAC score was categorized to asymptomatic, mild, moderate and severe symptoms. Individuals with radiological OA and ‘mild’ overall symptoms on the WOMAC score had reduced risk of falls compared to asymptomatic OA [OR: 0.402(0.172–0.940), p = 0.042]. Individuals with clinical OA and ‘severe’ overall symptoms had increased risk of falls compared to those with ‘mild’ OA [OR: 4.487(1.883–10.693), p = 0.005]. In individuals with radiological OA, mild symptoms appear protective of falls while those with clinical OA and severe symptoms have increased falls risk compared to those with mild symptoms. Both relationships between OA and falls were not mediated by physical limitations. Larger prospective studies are needed for further evaluation.

## Introduction

Approximately 30% of community-dwelling individuals over the age of 65 years have at least one fall per year [1, 2]. The risk of falls is increased further in institutionalized older adults. Falls are considered major public health problems in the geriatric population worldwide due to potentially serious consequences including hip fractures, traumatic brain injury, and debilitating long-term psychological impact. Falls risk in any individual increases with the cumulative presence of gait and balance disorders, skeletal muscle weakness and culprit medications [3]. Osteoarthritis (OA) has been considered an established risk factor for falls [4]. However, there is conflicting evidence surrounding the role of OA in falls [5].

Osteoarthritis is a disorder involving movable joints characterized by cell stress and extracellular matrix degradation initiated by micro- and macro-injury that activates maladaptive repair responses including pro-inflammatory pathways of innate immunity [6]. It is the most prevalent form of arthritis worldwide, characterized by mechanical joint pain and stiffness [5]. The prevalence of OA in Malaysia is 10–20%, with an estimated population of 30 million; similar to figures reported in the United States of America (USA) [7, 8]. Only a handful of studies have prospectively reviewed the incidence of falls in individuals with OA [5]. Furthermore, reported studies have employed highly varied definitions to diagnose the presence of OA, using either self-reported joint pain, clinical diagnosis of OA (self-reported and clinician-reported) or radiological diagnosis of OA. We found no published data directly linking physical impairment secondary to OA and falls. In a large prospective study over a 3-year period, presence of self-reported knee pain was significantly associated with falls, but radiographic evidence of OA was not [9]. Two small, uncontrolled studies that evaluated individuals with knee and hip OA found that fallers with OA were significantly more likely to have impaired physical function and balance [10, 11]. However, as these studies did not control for important factors such as established co-morbidities affecting OA, it was not possible to attribute the reduced physical functioning among their fallers to OA. Our main objectives were to investigate the association between OA and falls and the role of impaired physical function as a potential mediator of this association.

## Methods

### Ethics approval

The study was approved by the University Malaya Medical Centre Medical Ethics Committee (reference number: 925.4) and was compliant with the WMA Declaration of Helsinki 2013 [12]. Written, informed consent was obtained from all participants.

### Study design and baseline characteristics

This was a case-control, cross sectional sub-analysis of the baseline data obtained from a randomized controlled trial—the Malaysian Falls Assessment and Intervention Trial (MyFAIT) (trial registration number: ISRCTN11674947) [13], using the same participants with the same inclusion and exclusion criteria. Briefly, fallers (cases) were aged 65 years and above, with a history of high-risk falls in the past 12 months (i.e. two or more falls, or one injurious fall). Past history of falls was chosen as our main diagnostic approach as it is the strongest extrinsic risk factor for future falls [14]. Fallers were recruited from a teaching hospital’s geriatrics, primary care and specialty clinics, and the emergency department in Kuala Lumpur, Malaysia.

Non-fallers (controls) were aged 65 years and above with no history of falls. They were recruited from community centres within the hospital catchment area. Participants were excluded if they had at least one of the following criteria: (i) clinically diagnosed dementia (ICD-10 definition), (ii) severe physical disabilities (i.e. unable to walk even with a walking aid) or (iii) major psychiatric illness. Participant recruitment, interviews and physical assessments were performed in the same teaching hospital.

Basic demographics, anthropometrics, past medical history, comorbidities, self-reported falls history and medication data were collected from all participants. A fall was defined as “an unexpected event in which the participant comes to rest on the ground, floor or lower level” [15]. The operational definitions for OA diagnosis, severity of OA symptoms and physical function measures are described below.

### Diagnosis of osteoarthritis

All participants were assessed for presence of lower extremity knee or hip OA (i.e. either one or both sides). This was diagnosed using three methods: (i) self-report, (ii) clinician diagnosis or (iii) radiological diagnosis.

#### Self-reported OA

Participants were defined as having self-reported OA if they answered ‘yes’ to the following question, “Have you ever been told by a doctor that you have or have had knee or hip osteoarthritis?” [16].

#### Clinician diagnosed OA

The clinical diagnosis of OA was made by a rheumatologist blinded from clinical data and fall status, using both history and physical examination based on guidelines set by the European Project on Osteoarthritis (EPOSA) [16]. EPOSA incorporated the use of the Western Ontario and McMasters Universities Arthritis Index (WOMAC) [17] in their guidelines to identify presence of knee and hip OA. Knee OA was characterised by knee pain (evaluated by the WOMAC ‘pain’ subscale), plus any three of the following symptoms: (i) age over 50 years, (ii) morning stiffness (WOMAC ‘stiffness’ subscale), (iii) crepitus on active motion in at least one side, (iv) bony tenderness in at least one side, (iv) bony enlargement in at least one side, and (v) no palpable warmth of synovia in both knees [16]. Hip OA was characterised by hip pain (WOMAC ‘pain’ subscale) and all of the following symptoms: (i) age over 50 years, (ii) morning stiffness (WOMAC ‘stiffness’ subscale), and (iii) pain associated with hip internal rotation on at least one side. Presence of joint pain and stiffness was characterized by any score above zero for each subscale of the WOMAC index.

#### Radiological OA

Standard weight-bearing, anterior-posterior X-rays were taken from both sides of knees and hip for all participants. A radiologist blinded from clinical data assessed the radiographic images for severity of knee and hip OA using the Kellgren-Lawrence (KL) Grading Scale. This system uses an ordinal scale of: none (0), doubtful (1), minimal (2), moderate (3) and severe (4) [18]. KL grades 2–4 were defined in our study as ‘presence of radiological OA’, while grades 0 or 1 were considered ‘no radiological evidence of OA’.

### Severity of OA symptoms

If OA was identified using *any of the three* diagnostic methods, the severity of OA symptoms (subscale and categorized) was determined using the WOMAC index. Three language versions (English, Malay and Chinese) of the WOMAC index were provided to cater for the different ethnic groups of Malaysia.

#### WOMAC subscale scores

This 24-item questionnaire is presented in a visual analogue scale (VAS), 100 millimetre (mm) per item. It has three subscales: pain (5-item), stiffness (2-item) and function limitation (17-item); the maximum scores for each subscales are 500mm, 200mm and 1700mm respectively [17]. A maximum score indicates the highest levels of joint pain, stiffness and function limitation when performing activities of daily living.

#### Categorized scores

The total WOMAC Score is the summation scores of the three subscales. Using 25^th^, 50^th^ and 75^th^ percentile values as ordinal cut-offs, the total WOMAC Score was further categorized into 4 groups: no symptoms (0mm), mild symptoms (1-200mm), moderate symptoms (201-465mm) and severe symptoms (≥466mm).

### Physical function assessment

Functional reach and Timed Up and Go tests were demonstrated to the participant, followed by one trial run, before taking a second measurement for data. Shoes were kept on for these tests.

Functional reach (FR) is the maximal forward reach achieved. Each participant was instructed to stand upright with their left shoulder closest to a wall. A one-metre ruler was placed at shoulder (acromion) height, parallel to the floor. With the left arm raised to 90 degrees’ forward flexion, the start position of the participant’s outstretched fingers was taken at the fifth metacarpal head. Without contact with the wall, the participant was instructed to “reach out as far as you can without lifting your heels or moving your feet”. The distance of furthest reach was measured at the fifth metacarpal head. A maximal reach distance less than 18 centimetres (cm) indicated impaired physical function [19, 20].

The Timed Up and Go (TUG) test measures the time taken for the participant to complete a 3-metre continuous walk from and back to a seated position (46-centimetre-high chair with arms). Participants were instructed to walk independently at natural pace, and were allowed to use a walking aid. A completion time longer than 13.5 seconds (s) indicated impaired physical function [21].

### Statistical analysis

SPSS 20.0 (IBM SPSS Statistics) statistical software package was used for statistical analysis. Continuous data were expressed as mean (±standard deviation) or median (interquartile range). *Chi-square* test was used for comparison of categorical variables and independent *t-test* and *Mann-Whitney U* tests was used for comparison of continuous variables. Using cut-offs described earlier, continuous data were grouped into dichotomous variables. Binary logistic regression was used to determine the independent associated factors for falls after adjusted with significant demographic variables and physical performance. Dummy variables were created for comparisons between multiple categories, with the lowest category (no arthritis or mild arthritis) as the reference category. The strength of this association was presented in odds ratio (OR) and 95% confidence interval (CI).

## Results

A total of 389 participants (229 fallers, 160 non-fallers, mean age of 72.2±6.1 years, 67% females) were included for analysis. Radiological findings were available for 205 participants. Table 1 summarizes the baseline characteristics of fallers and non-fallers.

**Table 1:** Baseline characteristics in fallers and non-fallers.

Characteristics	n	Fallers (n = 229)	Non-Fallers (n = 160)	p-value
Age (years), mean (SD)	389	75.11 (7.11)	71.08 (5.22)	<0.001
Female sex, n (%)	389	150 (65.5)	113 (70.6)	0.288
BMI (kg/m^2^), mean (SD)	389	24.43 (3.83)	24.89 (4.15)	0.273
**Comorbidities, n (%)**				
*Heart diseases*	389	14 (6.1)	8 (5.0)	0.650
*Hypertension*	389	138 (60.3)	79 (49.7)	0.039
*Diabetes*	389	78 (34.1)	28 (17.6)	<0.001
*Stroke*	389	14 (6.1)	4 (2.5)	0.140
*Atrial fibrillation*	389	7 (3.1)	3 (1.9)	0.474
*Visual impairment*	389	74 (32.3)	33 (20.8)	0.012
**Type of Osteoarthritis, n (%)**				
*Self-reported OA*	389	88 (38.4)	51 (31.9)	0.184
*Clinical OA*	389	81 (35.4)	60 (37.5)	0.667
*Radiological OA*	205	106 (84.8)	65 (81.2)	0.505
*No Osteoarthritis*	389	67 (29.3)	55 (34.4)	0.284
**Physical Function**				
*TUG (s)*, *median (range)*	389	14.1 (6.0–100.0)	10.4 (5.1–40.0)	<0.001
*FR (cm)*, *mean (SD)*	389	24.4 (3.0–46.0)	28.9 (11.0–44.5)	<0.001
**Impaired Physical Function, n (%)**				
*TUG ≥13*.*5s*	389	122 (53.5)	32 (20.1)	<0.001
*FR ≤18cm*	389	63 (27.5)	15 (9.4)	<0.001

*Notes*: BMI = body mass index; OA = osteoarthritis; TUG = timed up and go; FR = functional reach.

Table 2 describes the associated factors for the occurrence of falls according to the type of OA diagnosis. The subgroup analysis showed that individuals with impaired physical function have about 3–5 fold increased risks of falls when in each of the three types of OA diagnosis. For individuals with diabetes and with visual impairment there were increased risks of falls in those with self-reported OA.

**Table 2:** Baseline odds ratios for the occurrence of falls according to type of osteoarthritis diagnosis.

	Falls, Odds Ratio (95% CI)		
	Radiological OA	Clinical OA	Self-reported OA
Number	171	141	139
Age	1.061 (0.982–1.146)	1.025 (0.947–1.108)	1.034 (0.963–1.111)
Gender	0.726 (0.356–1.480)	0.698 (0.319–1.524)	0.606 (0.256–1.435)
BMI	1.044 (0.946–1.151)	0.995 (0.904–1.096)	0.928 (0.841–1.024)
**Comorbidities**			
*Heart diseases*	1.918 (0.500–7.359)	1.301 (0.363–4.664)	0.762 (0.164–3.548)
*Hypertension*	1.061 (0.571–1.969)	1.620 (0.817–3.209)	1.328 (0.657–2.684)
*Diabetes*	1.730 (0.829–3.610)	1.673 (0.773–3.621)	5.793 (2.093–16.029)
*Stroke*	1.461 (0.364–5.862)	1.875 (0.351–10.014)	2.450 (0.500–12.011)
*Atrial fibrillation*	1.231 (0.109–13.849)	1.468 (0.130–16.583)	-
*Visual impairment*	1.595 (0.728–3.498)	1.119 (0.492–2.547)	3.082 (1.334–7.117)
**Impaired Physical Function**			
*TUG ≥13*.*5s*	4.000 (1.977–8.093)	3.579 (1.763–7.267)	4.971 (2.328–10.615)
*FR ≤18cm*	3.425 (1.408–8.331)	4.454 (1.796–11.044)	4.663 (1.520–14.305)

*Notes*: BMI = body mass index; OA = osteoarthritis; TUG = timed up and go; FR = functional reach; CI = confidence interval.

Table 3 summarizes the results of WOMAC severity of symptoms scores (subscale and categorized) and the total WOMAC score between fallers and non-fallers using different methods of diagnosis for OA. In both radiological and clinical OA, fallers had significantly higher joint stiffness and function limitation scores, and the total WOMAC score (i.e. maximum score indicates most pain, stiffness and function). Only fallers with clinical OA were seen to have higher joint pain scores compared to non-fallers. There were no differences in scores between fallers and non-fallers with self-reported OA for joint pain, stiffness and function limitation. Differences between categorized severity of symptoms were present between fallers and non-fallers for all diagnosis of OA. A greater proportion of fallers compared to non-fallers had severe OA symptoms in all types of OA. Using the predefined cut-offs to categorize the total WOMAC score into ordinal categories, severity of OA were then compared between fallers and non-fallers using logistic regression with dummy variables. Interestingly, among individuals radiological diagnoses of OA, fallers were significantly less likely to report mild OA symptoms that no OA symptoms. In individuals with clinical OA, all participants had at least mild OA symptoms. Fallers with clinical OA were significantly more likely to report severe OA symptoms compared to mild OA symptoms. No significant associations were observed between severity of symptoms and falls in self-reported OA.

**Table 3:** Comparison of WOMAC symptom severity scores between fallers and non-fallers according to osteoarthritis diagnosis.

	Radiological OA			Clinical OA			Self-reported OA		
	Fallers (n = 106)	Non-fallers (n = 65)	p-value	Fallers (n = 81)	Non-fallers (n = 60)	p-value	Fallers (n = 88)	Non-fallers (n = 51)	p-value
Subscale Scores (mm), median (IQR)*									
*Pain (5-item)*	40.0 (0–435)	20.0 (0–310)	0.228	100.0 (5–450)	50 (5–310)	0.003	50 (0–450)	30 (0–350)	0.156
*Stiffness (2-item)*	7.5 (0–200)	0 (0–200)	0.011	45 (0–200)	12.5 (0–200)	0.005	15.5 (0–200)	10 (0–200)	0.495
*Function Limitation (17-item)*	182.5 (0–1700)	48.0 (0–945)	0.010	327 (0–1700)	179 (0–945)	0.001	278 (0–1504)	150 (0–945)	0.061
Total WOMAC Score (mm), median (IQR) *	295 (0–1930)	88 (0–1245)	0.012	480.0 (10–2100)	282.5 (5–1245)	<0.001	361.5 (0–2100)	240 (0–1245)	0.068
Severity of Symptoms, n (%) **									
*No symptoms)*	27 (25.5)	16 (24.6)	reference	0	0	NA	15 (17.0)	7 (13.7)	reference
*Mild*	19 (17.9)	28 (43.1)	0.036	18 (22.2)	25 (41.7)	reference	17 (19.3)	18 (35.3)	0.150
*Moderate*	25 (23.6)	13 (20.0)	0.779	21 (25.9)	22 (36.7)	0.516	17 (19.3)	14 (27.5)	0.460
*Severe*	35 (33.0)	8 (12.3)	0.058	42 (51.9)	13 (21.7)	0.001	39 (44.3)	12 (23.5)	1.

*Notes*: OA = osteoarthritis; IQR = interquartile range; NA = not applicable.

* Mann-Whitney U.

** Logistic regression with dummy variables. Categorized using percentile cut-offs from Total WOMAC Score. “No symptoms”: 0mm. “Mild”: 1-200mm. “Moderate”: 201-465mm. “Severe”: ≥466mm.

To confirm these differences, Table 4 explored the association between falls and severity of OA symptoms according to type of OA, before and after three consecutive adjustments for confounders and impaired physical function. After adjustment, individuals with ‘mild’ OA symptoms in those with radiological OA (using the asymptomatic group as reference), were associated with reduced risk of falls. In participants with clinical OA (using ‘mild’ symptoms group as reference), individuals with ‘severe’ symptoms were associated with increased risk of falls. The ‘mild’ symptom group was used as reference as the minimum criteria for clinician diagnosed OA was joint pain. After adjustments for socio-demographic characteristics in Model 1, followed by additional adjustments of impaired TUG (Model 2) and FR (Model 3), both associations remained significant.

**Table 4:** Adjusted odds ratios for the occurrence of falls in different types of osteoarthritis.

	n	Severity of Symptoms *	Falls, Odds Ratio (95% CI)			
			Unadjusted	Model 1 **	Model 2 **	Model 3 **
Radiological OA	171	No symptoms (ref)	1	1	1	1
		Mild	0.402 (0.172–0.940)	0.382 (0.151–0.967)	0.369 (0.145–0.942)	0.361 (0.141–0.922)
		Moderate	1.140 (0.458–2.836)	0.978 (0.351–2.725)	0.862 (0.301–2.469)	1.011 (0.349–2.933)
		Severe	2.593 (0.967–6.950)	2.466 (0.845–7.193)	2.199 (0.702–6.886)	2.701 (0.860–8.488)
Clinical OA	141	No symptoms	-	-	-	-
		Mild (ref)	1	1	1	1
		Moderate	1.326 (0.566–3.106)	1.210 (0.486–3.015)	1.154 (0.459–2.901)	1.333 (0.511–3.476)
		Severe	4.487 (1.883–10.693)	3.685 (1.473–9.217)	3.345 (1.279–8.748)	3.213 (1.196–8.629)
Self-reported OA	139	No symptoms (ref)	1	1	1	1
		Mild	0.441 (0.144–1.345)	0.603 (0.151–2.403)	0.603 (0.150–2.415)	0.524 (0.127–2.166)
		Moderate	0.567 (0.181–1.776)	0.484 (0.116–2.009)	0.458 (0.109–1.932)	0.461 (0.108–1.973)
		Severe	1.517 (0.502–4.584)	2.208 (0.549–8.875)	1.917 (0.466–8.240)	1.361 (0.341–5.894)

*Notes*: OA = osteoarthritis; CI = confidence intervals.

* Categorized using percentile cut-offs from Total WOMAC Score. “No symptoms”: 0mm. “Mild”: 1-200mm. “Moderate”: 201-465mm. “Severe”: ≥466mm.

** Model 1 was adjusted for age, gender, ethnicity, comorbidities; Model 2 was additionally adjusted for impaired timed up and go; Model 3 was additionally adjusted for impaired functional reach.

## Discussions

In this study, lower extremity osteoarthritis was not associated with recurrent or injurious falls compared to individuals with no falls in the preceding 12 months. Impaired physical function was associated with increased risk of falls in all OA diagnosis. Radiological OA participants with ‘mild’ OA symptoms had a reduced risk of falls compared to individuals with no OA symptoms. Clinical OA participants with ‘severe’ OA symptoms had an increased risk of falls compared to individuals with ‘mild’ symptoms. These associations remained significant after adjustments for socio-demography, comorbidities, and impaired TUG and FR. This indicates that the association between osteoarthritis and falls is not directly attributable to impaired physical function.

### Relationship between falls and OA

As mentioned earlier, the evidence for increased risk of falls attributable to OA is conflicting [22]. A review of previously published studies has found that radiological evidence of OA is not associated with increased risk of falls. This is unsurprising considering that it is well established that radiological evidence of OA correlated poorly with physical symptoms of OA. However, the conflicting results of previous studies were also partly due to the highly varied criteria employed to determine the presence of OA.

In our study, no significant difference was observed in the proportion of individuals with either self-reported OA, clinical OA or radiological OA among fallers and non-fallers. However, when we considered the different degree of severity of OA symptoms, we found new knowledge on the potential reasons behind the mixed results on OA and falls. In agreement with previous studies, increased OA symptoms such pain, stiffness and physical function limitation were associated with falls [23]. However, such associations can only be found among the clinical OA subjects but not in the self-reported OA or radiological OA group. In contrast, in those with radiological OA, the mild OA symptom group had significantly lower falls risk compared to the asymptomatic group.

The results of our study have highlighted that fallers are not more likely to experience OA, regardless of the methods of detection. However, different methods of diagnosis revealed different interactions in the associations between severity of OA symptoms and the risk of falls. Our sample size is not adequately powered to detect smaller associations between OA and falls, and the convenience sampling employed in recruitment may not be representative of the general older population. The prevalence of self-reported clinician-diagnosed OA is nevertheless comparable to that reported in the USA population [24], while prevalence of joint pain in our control population is similar to that of rheumatic complaints among individuals aged ≥65 years in a Malaysian survey [25].

### Association between falls, OA severity symptoms and physical function

Physical performance in older adults with OA has been evaluated in a number of studies. However, these studies had rarely evaluated the specific relationship between the reduction in physical performance due to OA and falls [9, 26]. To the best of our knowledge, this was the first study to date describing the influence of impaired physical performance in the association between increased OA symptoms and falls using three different methods of detection. Alencar *et al* compared the functional mobility between osteoarthritic elderly women with and without a history of falls and found that fallers had significantly worse TUG scores but did not evaluate the symptom burden of their study population with OA [26].

We found that participants with mild OA symptoms were less likely to sustain recurrent or injurious falls when compared to the asymptomatic group in those with radiological OA, even after controlling for impaired physical performance and confounding variables. This suggested that impaired physical function was not the sole mediator of the association found. We postulated that subjects with mild OA symptoms may have more awareness of the presence of OA compared with those with asymptomatic radiological OA and became more careful or in their physical activity or perhaps restricted their activities of daily living. The asymptomatic radiological OA individuals could possibly be more mobile and may have had more exposure to risk of falls associated with greater levels of outdoor activity [27–29]. Similarly, among the clinical OA subjects, the increased risk of falls associated with severe joint symptoms remained significant even after adjustments for impaired TUG and FR performance, indicating that the association was not necessarily mediated by deficits in physical performance. It might be that other factors such as neuromuscular defects and central mechanisms are involved, where symptoms might interfere with cognition or executive function as suggested by the MOBILIZE study [30, 31].

### Strengths, limitations and recommendations

This study reveals previously unexposed relationships between falls, OA, and physical function. There are also few OA studies on at-risk fallers. Recruiting fallers from a higher risk group can be invaluable as at-risk fallers may have different physiology and psychological states as a result of their recurrent falls. As this was a cross-sectional study, cause-effect inferences cannot be drawn.

These findings should therefore be corroborated with larger, prospective studies as it will be a crucial step for establishing effective falls management strategies among older people with OA, where research evidence is scarce [32]. Future research should now be directed at understanding the differences in currently available OA diagnostic criteria and reported symptoms, as well as the reasons underlying the association between falls and joint symptoms but the lack of association with OA using currently employed diagnostic criteria. Future research should also determine the reasons behind the contradictory associations between decreased risks of falls among radiographic OA patients with mild OA symptoms.

In conclusion, radiological OA with mild overall symptoms measured with the WOMAC score may be protective of falls. Clinician-diagnosed OA can potentially flag at-risk individuals for falls if their overall symptoms on the WOMAC score are severe. However, self-reporting as an OA diagnostic tool may not be suitable in studies involving falls subjects. Impaired physical function is not a mediator to the association between osteoarthritis and falls, which raises the possibility of the role of psychological and cognitive factors in this relationship.
